# The Impact of a Horse Kick: A Systematic Review of Horse-Related Maxillofacial Trauma

**DOI:** 10.3390/jcm15166189

**Published:** 2026-08-10

**Authors:** Luigi Angelo Vaira, Hareem Qadeer, Sebastiano Stellino, Jerome R. Lechien, Antonino Maniaci, Fabio Maglitto, Giuseppe Consorti, Giulio Cirignaco, Łukasz Woźniak, Carlos Navarro-Cuéllar, Bożena Antonowicz, Jan Borys, Giovanni Salzano, Giacomo De Riu

**Affiliations:** 1 Maxillofacial Surgery Operative Unit, Department of Medicine, Surgery and Pharmacy, University of Sassari, 07100 Sassari, Italy; h.qadeerahmad@studenti.uniss.it (H.Q.); sebastiano.stellino@hotmail.it (S.S.); gderiu@uniss.it (G.D.R.); 2 PhD School of Biomedical Sciences, Department of Biomedical Science, University of Sassari, 07100 Sassari, Italy; 3 Department of Surgery, Mons School of Medicine, UMONS Research Institute for Health Sciences and Technology, University of Mons (UMons), 7000 Mons, Belgium; jerome.lechien@umons.ac.be; 4 Department of Otolaryngology-Head Neck Surgery, Elsan Polyclinic of Poitiers, 86000 Poitiers, France; 5 Department of Medicine and Surgery, University of Enna Kore, 94100 Enna, Italy; antonino.maniaci@unikore.it; 6 Head and Neck Section, Department of Neurosciences, Reproductive and Odontostomatological Science, Federico II University of Naples, 80131 Naples, Italy; fabio.maglitto@unina.it (F.M.); giovanni.salzano@unina.it (G.S.); 7 Division of Maxillofacial Surgery, Department of Neurological Sciences, Marche University Hospitals—Umberto I, 60100 Ancona, Italy; giuseppe.consorti@ospedaliriuniti.marche.it (G.C.); giuliocirignaco@gmail.com (G.C.); 8 Department of Biomedical Sciences and Public Health, Polytechnic University of Marche, 60121 Ancona, Italy; 9 Department of Medicine, Section of Maxillo-Facial Surgery, University of Siena, Viale Bracci, 53100 Siena, Italy; 10 Department of Dental Surgery, Medical University of Bialystok, 15-276 Bialystok, Poland; lukasz.wozniak@umb.edu.pl (Ł.W.); bozena.antonowicz@umb.edu.pl (B.A.); 11 Maxillofacial Surgery Department, Hospital Gregorio Marañon, Universidad Complutense de Madrid, 28040 Madrid, Spain; canava03@ucm.es; 12 Department of Maxillofacial and Plastic Surgery, Medical University of Bialystok, 15-276 Bialystok, Poland; jan.borys@umb.edu.pl

**Keywords:** horse kick, equestrian injuries, maxillofacial trauma, facial fractures, orbital trauma

## Abstract

**Objectives**: To systematically review the evidence on horse-related maxillofacial trauma, with emphasis on horse-kick injuries, anatomical patterns, associated injuries, management, outcomes, and prevention. **Methods**: This systematic review followed the PRISMA 2020 statement. PubMed/MEDLINE, Scopus, Web of Science, and the Cochrane Library were searched for original clinical studies reporting facial, craniofacial, orbital, dental, or maxillofacial injuries related to horse-associated activities. Data were extracted on study characteristics, demographics, mechanism, mounted/unmounted status, anatomical site, associated injuries, treatment, complications, outcomes, mortality, and protective equipment. Due to substantial heterogeneity in study design, denominators, and outcome reporting, a structured narrative synthesis was performed without formal meta-analysis. **Results**: Twenty-eight original clinical studies were included. Maxillofacial-specific series consistently identified horse kicks as a major mechanism, especially among unmounted individuals. Injuries most frequently involved the midface, orbit, zygomaticomaxillary complex, mandible, nasal region, and dentoalveolar structures. Severe presentations included optic nerve avulsion, orbitocranial penetrating trauma, naso-orbito-ethmoid and frontal sinus fractures, airway compromise, intracranial injury, and multisystem trauma. Management ranged from conservative treatment to operative fixation, orbital reconstruction, soft tissue repair, advanced airway management, and multidisciplinary care. Helmet use was often absent or poorly documented, particularly during unmounted activities. **Conclusions**: Horse kicks represent a distinct localized high-energy mechanism of maxillofacial trauma. Prevention should extend beyond riding and include facial protection, protective eyewear, and safety protocols for ground-based horse handling.

## 1. Introduction

Equestrian activities are widely practiced worldwide for recreational, occupational, and competitive purposes. Despite their popularity, interactions with horses are associated with a substantial risk of injury because of the animal’s size, strength, speed, and unpredictable behavior. Horse-related trauma may involve the head, spine, thorax, abdomen, extremities, and craniofacial region, with patterns comparable in severity to those observed in other high-energy mechanisms [1,2,3]. Although falls from horseback are the most frequently reported mechanism in general equestrian trauma, injuries sustained while unmounted, particularly during grooming, feeding, handling, shoeing, or working near the animal, represent an important and often underestimated subgroup [4,5,6].

The maxillofacial region is particularly vulnerable in horse-related trauma. Conventional riding helmets primarily protect the cranial vault but leave the facial skeleton, orbit, jaws, and dentoalveolar structures largely exposed. More broadly, maxillofacial fracture patterns are influenced by the mechanism of injury, direction and magnitude of impact, patient-related factors, and the activity being performed at the time of trauma; this has been observed across several traumatic settings, including road traffic accidents, assaults, falls, sports-related injuries, and occupational trauma [7,8,9,10]. In horse-related facial trauma, previous series have frequently reported involvement of the midface, orbit, zygomaticomaxillary complex, mandible, nasal bones, and dentoalveolar region [5,11,12,13,14,15].

Among horse-related mechanisms, the hoof kick appears to represent a distinct high-energy event. Unlike falls from horseback, which often produce multisystem trauma, horse kicks typically occur in unmounted individuals and deliver a concentrated force to a limited anatomical area. Several studies have consistently associated kicks with facial fractures and complex midfacial injuries, particularly involving the orbit, zygoma, central midface, and mandible [4,5,6,11,13,14,15,16].

**Figure 1:**
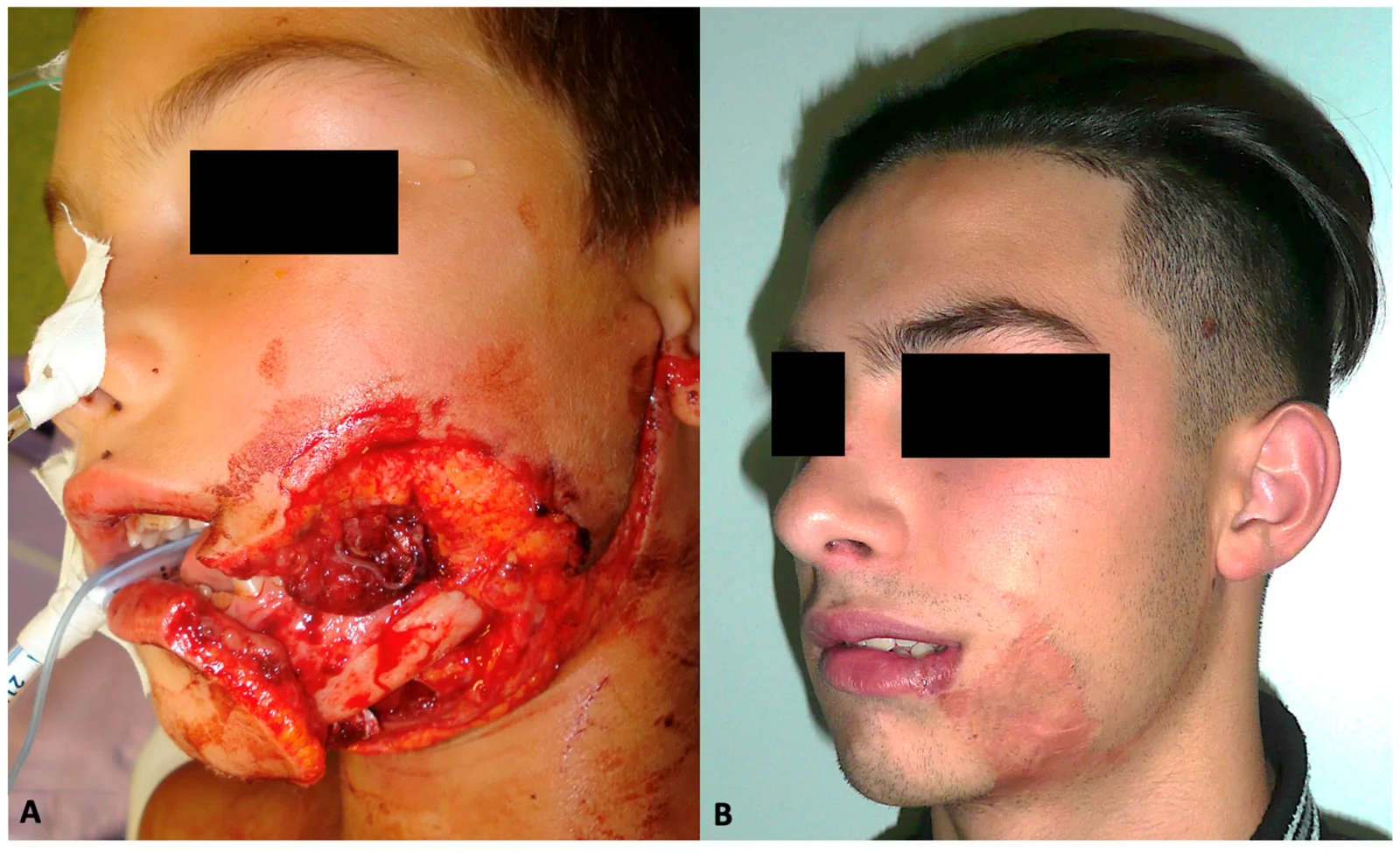
Severe horse-kick soft tissue injury in a 7-year-old child. (**A**): Initial clinical presentation showing extensive full-thickness laceration and avulsive soft tissue loss of the lower facial region, with exposure of the underlying tissues and contamination of the wound. (**B**): Seven-year follow-up after surgical repair, showing satisfactory facial contour and soft tissue healing, with residual scarring of the lower lip and chin region.

The clinical spectrum ranges from isolated soft tissue lacerations [Figure 1] or mandibular fractures to severe craniofacial injuries requiring multidisciplinary management. Reported severe injuries include naso-orbito-ethmoid fractures with frontal sinus involvement, orbitocranial penetrating trauma, traumatic optic nerve avulsion, and major midfacial disruption requiring advanced airway management [17,18,19,20]. These reports highlight the need for early recognition, appropriate imaging, ophthalmological and neurosurgical assessment when indicated, and careful surgical planning.

Despite its clinical relevance, the literature remains fragmented. Many studies address equestrian trauma in general, pediatric injuries, neurological trauma, occupational injuries, or animal-related facial fractures, whereas fewer focus specifically on horse-related maxillofacial trauma. In addition, available reports differ in design, inclusion criteria, denominators, injury definitions, and outcome reporting, limiting the possibility of direct comparisons across studies. Therefore, this systematic review aimed to synthesize the available evidence on horse-related maxillofacial trauma, with particular emphasis on horse-kick injuries, focusing on epidemiology, mechanisms of injury, mounted versus unmounted activity, anatomical distribution, associated injuries, management, outcomes, and preventive implications.

## 2. Materials and Methods

This systematic review was designed and conducted in accordance with the PRISMA 2020 statement [21]. The review question was structured according to a PECO framework, as the review focused on an exposure rather than on a therapeutic intervention. The population of interest included human patients with facial, craniofacial, orbital, dental, or maxillofacial trauma. The exposure was horse-related trauma, with particular emphasis on horse-kick injuries. When available, comparators included different mechanisms of injury, mounted versus unmounted activity, use versus non-use of protective equipment, and operative versus non-operative management. Outcomes of interest included injury mechanism, anatomical distribution, associated injuries, treatment, complications, length of hospital stay, functional or cosmetic outcomes, and mortality. A completed PRISMA 2020 checklist is provided as Supplementary Document S2.

A comprehensive electronic search was conducted between 2 and 3 May 2026 in PubMed/MEDLINE, Scopus, Web of Science Core Collection, and the Cochrane Library. For each database, the search was structured around two main concept blocks: (1) horse- and equestrian-related exposure terms and (2) cranio-maxillofacial injury terms. Synonyms and related terms within each concept block were combined using the Boolean operator OR, whereas the two concept blocks were combined using the Boolean operator AND. Controlled vocabulary was used where available, including MeSH terms in PubMed/MEDLINE and the Cochrane Library, together with database-specific free-text field codes. Free-text terms were searched in the title and abstract fields in PubMed/MEDLINE, in TITLE-ABS-KEY fields in Scopus, in the Topic field in Web of Science, and in title, abstract, and keyword fields in the Cochrane Library. Truncation symbols were used to retrieve relevant word variants. No restrictions were applied regarding patient age or sex. The complete reproducible search strategy for each electronic database is reported in Supplementary Table S1. Reference lists of the included articles were also screened manually to identify additional eligible reports.

Studies were eligible if they reported original clinical data on human patients with facial, craniofacial, orbital, dental, or maxillofacial injuries related to horse-associated activities. Both mounted and unmounted equestrians were considered. Eligible mechanisms included horse kicks, hoof-related impacts, falls from horseback, trampling, crushing injuries, head-butting, biting, and other direct horse-related trauma. Case reports, case series, retrospective and prospective observational studies, cohort studies, registry-based studies, and trauma database studies were eligible when they provided extractable clinical information relevant to the objectives of the review.

Studies were excluded if they did not involve human patients, were veterinary, cadaveric, experimental, or purely biomechanical, or did not report facial or maxillofacial trauma. Reviews, editorials, letters without original clinical data, conference abstracts with insufficient information, and duplicate publications were also excluded. General equestrian trauma studies were included only when craniofacial, facial, orbital, dental, or maxillofacial data were extractable or clinically relevant. Studies reporting broad horse-related trauma without separable maxillofacial outcomes were retained only for contextual discussion and were not included in quantitative extraction.

After duplicate removal, titles and abstracts were screened independently by two reviewers. Full texts of potentially eligible articles were then retrieved and assessed for inclusion. Disagreements were resolved through discussion and consensus, with consultation of a third reviewer when necessary. The study selection process was summarized using a PRISMA flow diagram, including the number of records identified, screened, excluded, assessed in full text, and finally included in the qualitative synthesis.

Data were extracted using a standardized form designed for this review. Extracted variables included first author, year, country, study design, study period, sample size, patient demographics, injury mechanism, mounted or unmounted status, protective equipment use, type and site of maxillofacial injury, associated injuries, imaging methods, treatment strategy, surgical management, complications, length of stay, functional or cosmetic outcomes, mortality, and main findings. Particular attention was given to variables potentially suitable for descriptive comparison, including sex distribution, age, proportion of kick-related injuries, proportion of unmounted injuries, fracture distribution, surgical treatment rate, associated injury rate, head or intracranial injury rate, mortality, and protective equipment documentation.

Because the literature was heterogeneous, studies were classified according to their main contribution. Maxillofacial-specific retrospective series and horse-kick-focused studies were considered core studies for data extraction. Studies addressing ocular, orbital, airway, neurological, pediatric, occupational, or broader equestrian trauma were included when they provided relevant data or clinically important insights. Case reports were extracted separately and used to describe rare but severe injury patterns, including optic nerve avulsion, naso-orbito-ethmoid fractures, frontal sinus involvement, orbitocranial penetrating trauma, and complex airway management.

Methodological quality was assessed according to study design. Observational, retrospective cohort, and registry-based studies were evaluated using the Newcastle–Ottawa Scale or an adapted version suitable for non-randomized studies [22]. Case reports and case series were assessed using the Joanna Briggs Institute critical appraisal tools [23]. Risk of bias assessment was performed independently by two reviewers, and disagreements were resolved by consensus. The assessment considered patient selection, exposure and mechanism reporting, outcome reporting, follow-up, comparability, and overall methodological quality.

A structured narrative synthesis was performed. Studies were summarized according to mechanism of injury, mounted or unmounted status, anatomical distribution, associated trauma, treatment strategy, outcomes, and preventive implications. No formal meta-analysis was performed because of substantial heterogeneity across studies, including differences in denominators, reporting units, inclusion criteria, and outcome definitions. Descriptive quantitative data were reported at the study level when available, with caution in interpreting fracture frequencies because some studies reported data per patient, others per fracture, and others per injury subunit.

The certainty of evidence was assessed using the GRADE approach where applicable, considering study limitations, inconsistency, indirectness, imprecision, and potential publication bias [24]. Given that most available evidence consisted of retrospective studies, case series, registry-based analyses, and case reports, the overall certainty of evidence was expected to be low to moderate. Publication bias and selective reporting were considered qualitatively by comparing reported methods, outcomes, and completeness of extracted data across included studies.

## 3. Results

**Figure 2:**
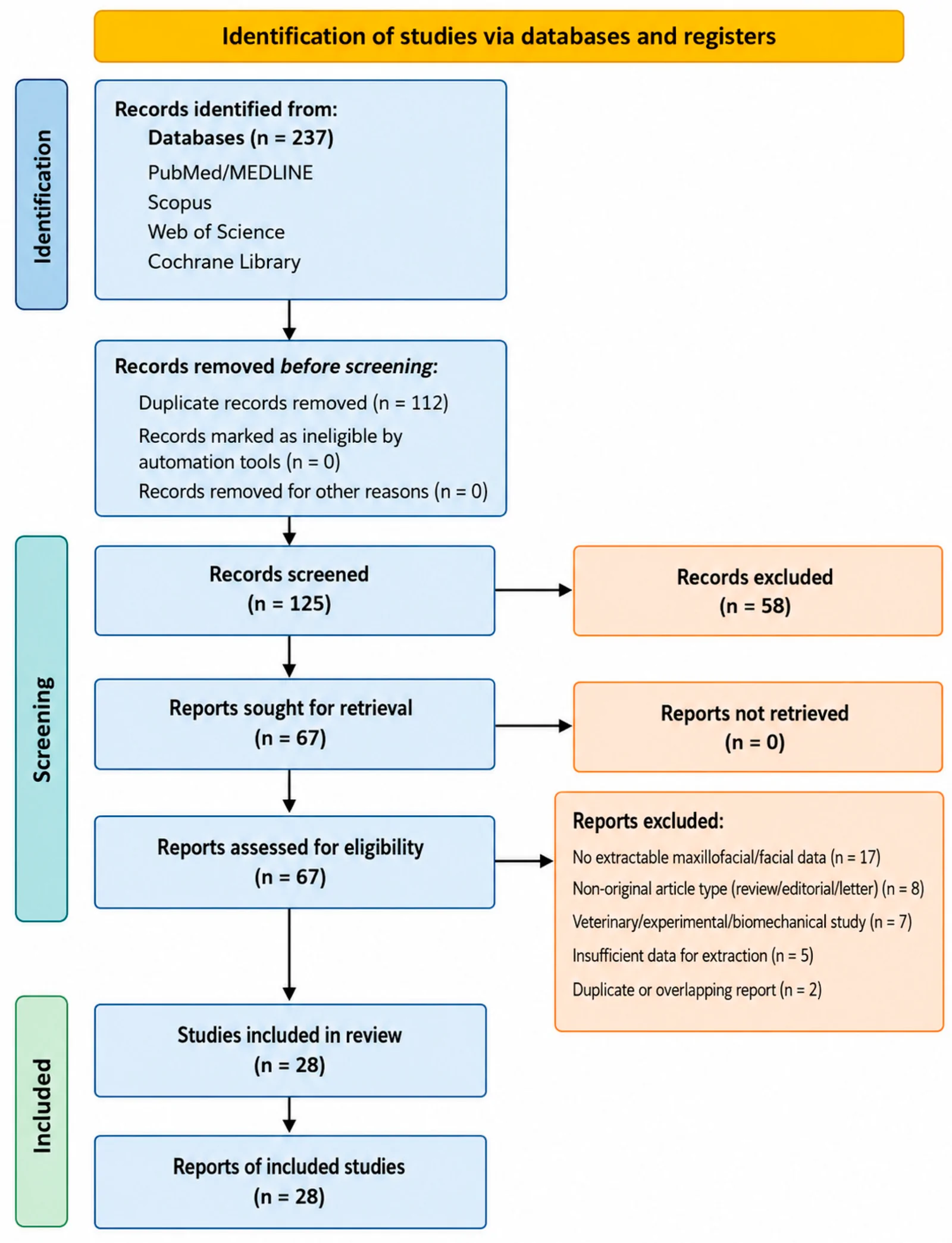
PRISMA flow diagram of the study selection process.

The database search identified 237 records. After duplicate removal, 125 records were screened by title and abstract, and 67 full-text articles were assessed for eligibility. Thirty-nine articles were excluded after full-text assessment, mainly because they did not provide separable maxillofacial, facial, craniofacial, orbital, or dental data, did not report original clinical data, were veterinary or experimental studies, or provided insufficient information for extraction. Twenty-eight original clinical studies were included in the qualitative synthesis [1,2,4,5,6,11,12,13,14,15,16,17,18,19,20,25,26,27,28,29,30,31,32,33,34,35,36,37]. Because of methodological and clinical heterogeneity, no formal meta-analysis was performed. The study selection process is summarized in Figure 2.

### 3.1. Study Characteristics

The included studies were heterogeneous in design, setting, and outcome reporting. Most were retrospective single-center or registry-based studies, while only a few were prospective, multicenter, or survey-based. The core evidence base consisted of maxillofacial-specific series reporting horse-related or equine-associated facial injuries and fractures [5,6,11,12,13,14,15]. Additional studies provided relevant data from broader equestrian trauma cohorts, occupational or farm-related populations, pediatric series, ocular/orbital trauma studies, and severe case reports. The main characteristics of the included studies are summarized in Supplementary Table S2.

### 3.2. Risk of Bias

**Figure 3:**
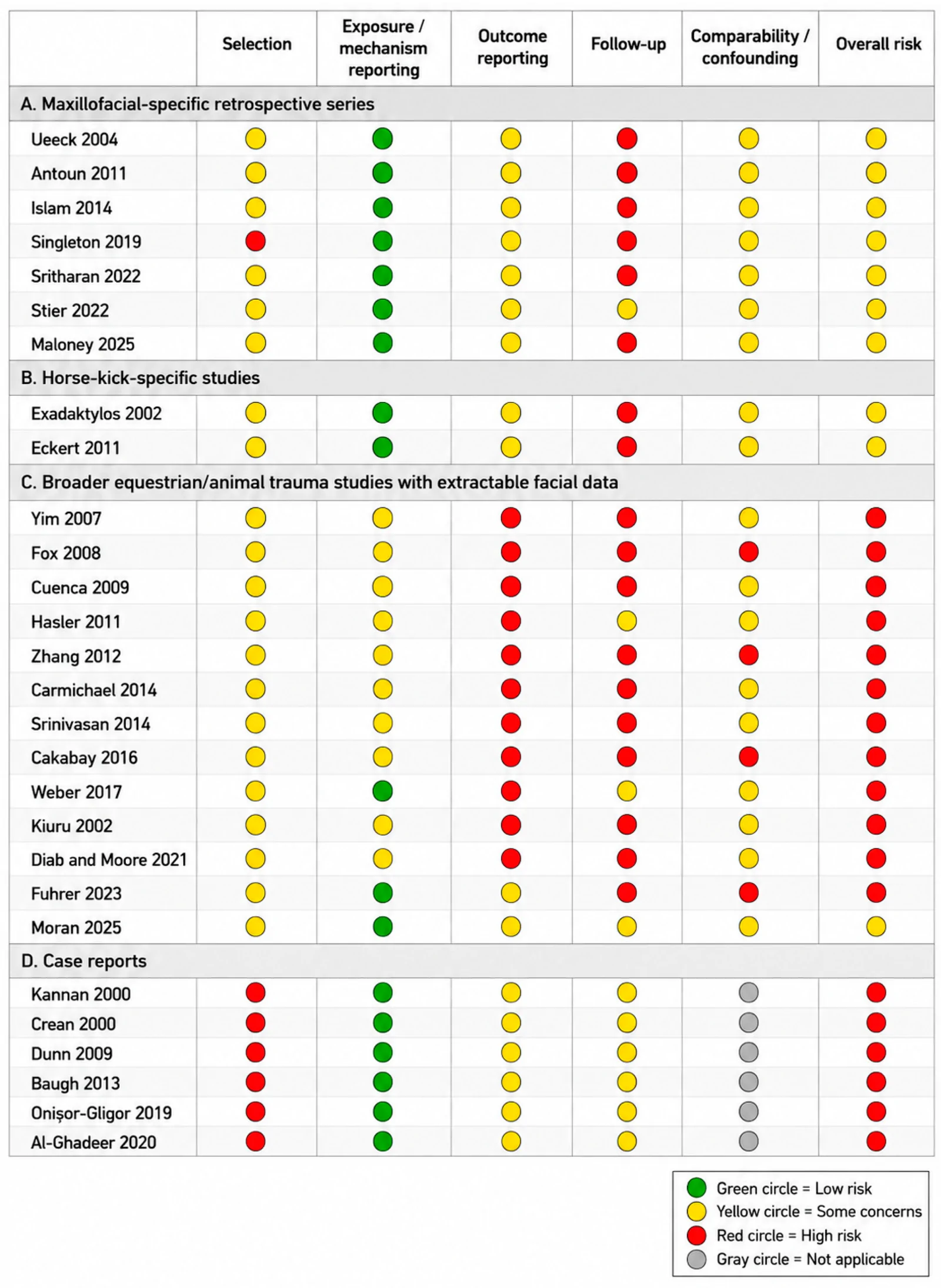
Risk of bias and methodological quality assessment of the included studies [1,2,4,5,6,11,12,13,14,15,16,17,18,19,20,25,26,27,28,29,30,31,32,33,34,35,36,37].

The methodological quality of the included studies varied considerably. The strongest evidence came from retrospective maxillofacial series and registry-based studies with clearly reported mechanisms and anatomical fracture patterns. However, most studies were limited by retrospective design, small sample size, heterogeneous outcome definitions, inconsistent denominators, and incomplete reporting of protective equipment, follow-up, complications, and long-term functional or cosmetic outcomes. Operative-only cohorts and trauma-center-based series were considered at risk of selection bias because they may overrepresent more severe injuries. Broader equestrian trauma studies provided useful contextual information but were often affected by indirectness, as facial injuries were sometimes grouped with head, cervical spine, or ocular trauma. Case reports were considered clinically informative but at high risk of publication and selection bias. Overall, the certainty of evidence was low to moderate. The methodological quality assessment is summarized in Figure 3.

### 3.3. Patient Demographics

Patient demographics varied according to study setting and population. Most maxillofacial-specific series showed a predominance of female patients and young to middle-aged adults. Female predominance was reported by Ueeck et al. [11], Antoun et al. [5], Islam et al. [12], Singleton et al. [13], Sritharan et al. [14], and Stier et al. [6], with mean or median ages generally ranging from the late twenties to the late thirties. However, this pattern was not universal. Maloney et al. [15] reported a slight male predominance, while Moran et al. [37], in an ocular/adnexal trauma cohort, reported more males than females. Broader and occupational cohorts also differed: Yim et al. [27], studying professional Jockey Club workers, reported a clear male predominance. Specific subgroups included children [20,25,29], farmers [34], professional equestrian workers [27], and farriers or hoof care practitioners [36].

### 3.4. Anatomical Distribution of Maxillofacial Injuries

**Figure 4:**
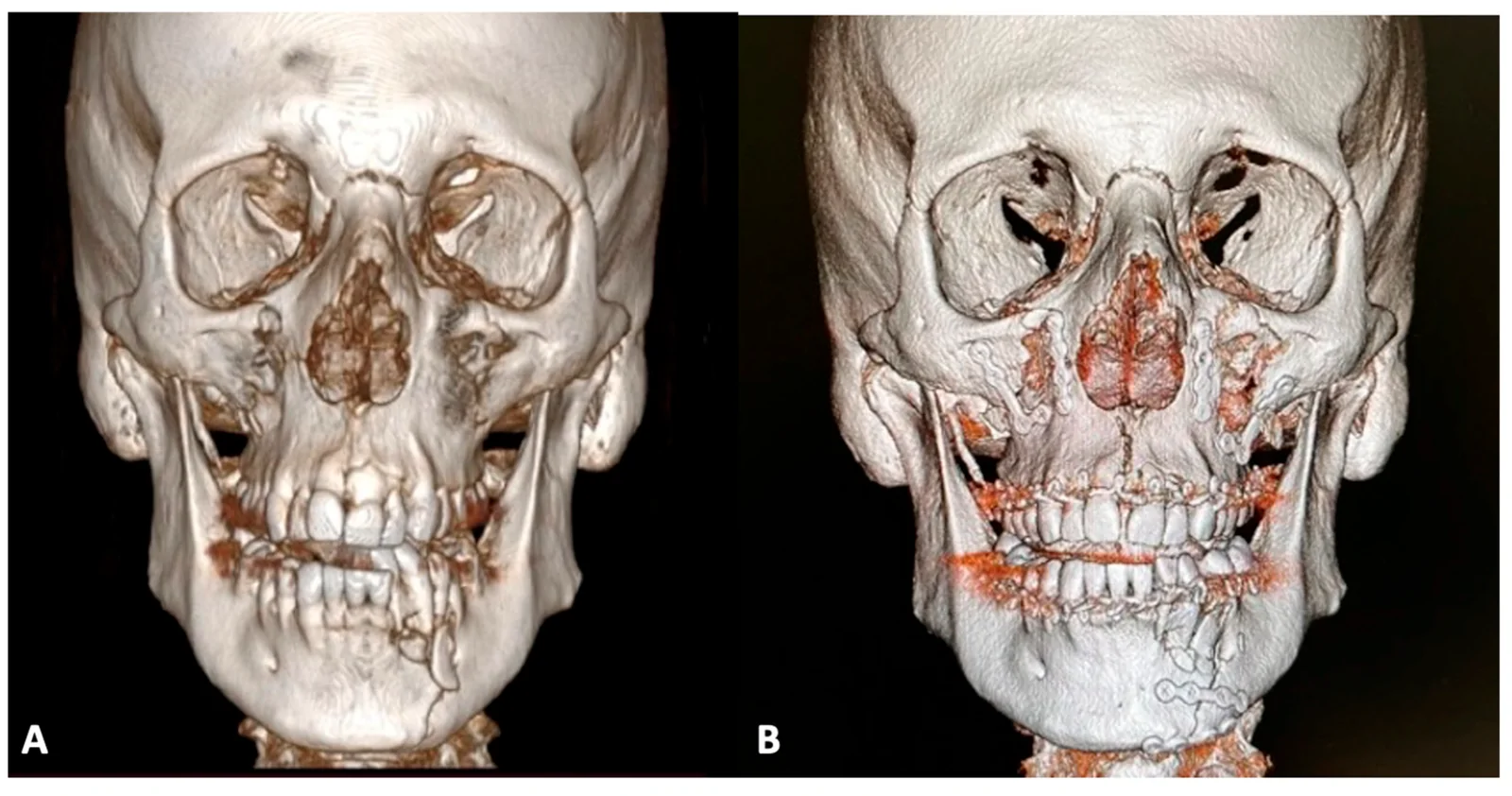
Horse-kick-related maxillofacial fractures involving the mandible and midface. (**A**): Preoperative three-dimensional CT reconstruction showing a left parasymphyseal mandibular fracture associated with dentoalveolar trauma and a Le Fort I-type fracture of the maxilla. (**B**): Postoperative three-dimensional CT reconstruction after open reduction and internal fixation, showing restoration of mandibular and maxillary continuity.

Although denominators varied across studies, a recurrent anatomical pattern emerged. Horse-related maxillofacial trauma, particularly after kicks, most frequently involved the midface, orbit, zygomaticomaxillary complex, mandible, nasal bones, and dentoalveolar region [Figure 4].

Orbital involvement was common and clinically important. Orbital fractures were reported by Antoun et al. [5], Islam et al. [12], Singleton et al. [13], Sritharan et al. [14], and Maloney et al. [15]. Moran et al. [37], in an ocular/adnexal trauma series, reported orbital fractures as the most common injury, accounting for 42 of 73 ocular injuries. Severe ocular outcomes included optic nerve avulsion with permanent visual loss [20] and orbitocranial penetrating trauma with superior orbital fissure syndrome [18].

The zygoma, zygomaticomaxillary complex, and midface were also frequently involved. Antoun et al. [5] reported zygomatic fractures in 22 of 35 unmounted equestrians, while Maloney et al. [15] found zygomaticomaxillary complex fractures to be the most common pattern. Ueeck et al. [11] reported that the midface was the most frequently affected region, and Stier et al. [6] found frequent involvement of the orbit, maxilla, zygoma, nose, and mandible.

Mandibular and dentoalveolar injuries were less consistently reported but remained clinically relevant. Mandibular fractures were described by Antoun et al. [5], Islam et al. [12], Singleton et al. [13], Sritharan et al. [14], and Stier et al. [6]. Dentoalveolar injuries were reported in several maxillofacial series and were also relevant in occupational settings, particularly among farriers and hoof care practitioners [36]. Pediatric mandibular trauma was illustrated by Crean et al. [25], who reported bilateral mandibular fractures in a 2-year-old child managed conservatively.

Naso-orbito-ethmoid and frontal sinus injuries were less frequent but represented some of the most severe patterns. These were reported in the series by Antoun et al. [5], Islam et al. [12], Singleton et al. [13], Sritharan et al. [14], and Maloney et al. [15]. Onișor-Gligor et al. [19] described a severe horse-kick injury causing a type II naso-orbito-ethmoid fracture with bilateral anterior and posterior frontal sinus wall fractures, neurological deterioration, and need for intensive care and cranio-maxillofacial management. Soft tissue injuries, including complex lacerations and contaminated wounds, were frequent in Ueeck et al. [11], Exadaktylos et al. [4], Singleton et al. [13], and Sritharan et al. [14].

### 3.5. Associated Injuries

Associated injuries were variably reported but were clinically important. Head and intracranial injuries were among the most relevant. Ueeck et al. [11] reported associated injuries in 46 of 62 patients, most commonly involving the head and upper extremity. Sritharan et al. [14] reported non-isolated maxillofacial trauma in 15 of 51 patients, including 10 head injuries and 2 cervical spine fractures, with one death due to intracranial trauma. Maloney et al. [15] reported head and spinal injuries in 4 patients each and found an association between frontal bone fracture and head injury.

Ocular and orbital-associated injuries were prominent. Moran et al. [37] reported multiple ocular/adnexal injuries in 15 of 50 patients and intracranial hemorrhage associated with orbital fracture in 9 patients. Their cohort included traumatic optic neuropathy, open globe injuries, retrobulbar hemorrhage, hyphema, commotio retinae, corneal abrasions, eyelid lacerations, and orbital fractures. Severe case reports confirmed that horse-kick trauma may cause permanent visual loss or occult orbitocranial extension even when external injury appears limited [18,20].

Broader trauma cohorts also reported cervical spine, thoracic, abdominal, pelvic, and orthopedic injuries. Kiuru et al. [26] reported spinal injuries in 13 of 46 CT-assessed patients, while Weber et al. [2] found that falls were associated with head and spinal injuries and crush injuries with pelvic and abdominal trauma. Yim et al. [27] and Cuenca et al. [29] also reported frequent multisystem injuries in occupational and pediatric cohorts. Airway compromise was rarely quantified but was highlighted by Kannan et al. [17], who described advanced airway management in severe midfacial disruption after a horse kick.

### 3.6. Management, Outcomes, and Complications

Management ranged from conservative treatment to complex operative and multidisciplinary care. Conservative management was used in selected minimally displaced fractures and pediatric injuries. Antoun et al. [5] reported conservative management in 13 of 35 unmounted equestrians, and Crean et al. [25] described successful non-operative treatment of bilateral mandibular fractures in a 2-year-old child. Observation or conservative follow-up was also reported in selected ocular injuries when surgical correction was not indicated or feasible [20].

Surgical treatment was common in maxillofacial-specific series, especially for displaced fractures, orbital involvement, mandibular fractures, naso-orbito-ethmoid injuries, and complex soft tissue lacerations. Antoun et al. [5] reported active treatment in approximately 60% of patients. Singleton et al. [13], an operative-only cohort, reported operative management of 54 of 61 fractures, mostly with internal fixation. Sritharan et al. [14] reported 59 operatively managed injuries among 95 recorded injuries, and Diab and Moore [35] reported an overall operative rate of 56% in animal-related facial fractures. Ocular and orbital injuries often required specialist management; Moran et al. [37] reported surgery in 16 of 50 patients, with a higher surgical rate among kick-related injuries.

Outcomes and complications were inconsistently reported. Ocular morbidity was among the most important long-term sequelae, including strabismus, lacrimal obstruction, ptosis, eyelid malposition, retinal detachment, optic atrophy, corneal scarring, and loss of the eye [37]. Permanent visual loss was reported after traumatic optic nerve avulsion [20]. Neurological morbidity was described in severe cases and broader trauma cohorts, including cranial nerve palsy, vertigo, dysphagia, supraorbital neuralgia, paraplegia, and quadriplegia [30,32]. Mortality was uncommon in maxillofacial-specific series but was reported in selected cohorts, usually related to severe head injury or multisystem trauma [1,11,14,26].

### 3.7. Preventive Measures and Protective Equipment

Preventive measures were frequently discussed but inconsistently documented. Helmet use was often absent or poorly recorded. Ueeck et al. [11] reported that 50 of 62 patients were not wearing helmets, Carmichael et al. [1] confirmed helmet use in only 12 of 284 admitted patients, and Eckert et al. [16] reported helmet use in only 1 patient in the uploaded preliminary hoof-kick cohort. Maloney et al. [15] reported overall helmet compliance of 35%, with a marked difference between mounted and unmounted patients: 62% versus 10%, respectively.

Several studies emphasized that standard helmets protect the cranial vault but not the facial skeleton. Exadaktylos et al. [4] recommended additional face shields after observing a high rate of isolated facial injuries in unmounted hoof-kick patients. Antoun et al. [5], Stier et al. [6], and Maloney et al. [15] similarly discussed the need for improved facial protection. Occupational groups may require specific preventive strategies: Fuhrer et al. [36] reported frequent head, face, and dental injuries among farriers and hoof care practitioners and highlighted eye protection and dental first aid as important preventive measures. Overall, the evidence suggests that prevention should extend beyond riding and include safety education, facial protection, protective eyewear, and clear protocols for unmounted activities such as grooming, feeding, handling, shoeing, and working near horses.

## 4. Discussion

This systematic review identifies horse-related maxillofacial trauma as a distinct and clinically relevant subset of equestrian injuries. Across heterogeneous study designs and clinical settings, the most consistent pattern was the association between direct horse kicks, unmounted activity, and concentrated injury to the facial skeleton. Rather than representing merely one mechanism within broader equestrian trauma, the hoof kick appears to produce a recognizable clinical phenotype characterized by localized high-energy transfer, frequent midfacial and orbital involvement, and the potential for severe ocular, neurological, airway, and soft tissue complications.

The recurrent involvement of the orbit, zygomaticomaxillary complex, central midface, mandible, and dentoalveolar region is unlikely to be incidental. It probably reflects the concentrated transfer of energy from the hoof to a relatively small and anatomically exposed facial surface. Orbital involvement is particularly important because the functional consequences may be disproportionate to the apparent external injury, and severe visual impairment may occur even in the absence of major globe disruption. These findings support a low threshold for detailed ophthalmological assessment and cross-sectional imaging after direct impact to the upper or central face.

The distinction between mounted and unmounted trauma also has important clinical and preventive implications. Falls from horseback more commonly expose the entire body to deceleration and multisystem injury, whereas ground-based interactions place the face within the trajectory of a direct kick. This difference may explain why unmounted patients can sustain severe and relatively localized craniofacial trauma despite the absence of a fall. It also highlights a gap in traditional prevention strategies, which remain largely centered on riding rather than on horse handling, grooming, feeding, and occupational exposure.

### 4.1. Horse Kicks as a Distinct High-Energy Mechanism

The mechanism of injury is essential for understanding horse-related maxillofacial trauma. A fall from horseback usually involves deceleration, impact with the ground, or collision with surrounding structures. By contrast, a horse kick is a direct, localized, high-energy blunt impact delivered by the hoof. This mechanism explains why kicks frequently cause concentrated facial skeletal injuries, particularly to the midface, orbit, zygoma, and mandible.

The consistency of this pattern across different countries, clinical settings, and study designs supports the interpretation of the hoof kick as the defining mechanism of equine-related facial trauma. Its clinical significance lies not only in the frequency of fractures, but also in the concentration of force within anatomically vulnerable regions. This may account for the occurrence of complex midfacial disruption, naso-orbito-ethmoid and frontal sinus fractures, orbital apex injury, optic nerve damage, penetrating craniofacial trauma, and difficult airway scenarios. Although these severe presentations represent the upper end of the injury spectrum, they justify a mechanism-driven approach and a high index of suspicion even when the external wound appears limited.

### 4.2. Midface and Orbital Vulnerability

The midface and orbit appear to represent the principal anatomical vulnerability zones in horse-kick trauma. Their prominence and limited capacity to dissipate a concentrated external force may explain the recurrent involvement of the zygomaticomaxillary complex, orbital walls, maxilla, and nasal region. From a functional perspective, orbital injury deserves particular attention because visual loss, diplopia, ocular motility disorders, lacrimal dysfunction, and optic nerve injury may persist long after skeletal healing. Consequently, the clinical relevance of orbital trauma should be assessed not only in terms of fracture pattern, but also according to its potential visual and neurological consequences.

These findings have practical implications. Any patient with horse-kick trauma to the orbit, zygoma, central midface, or frontal region should undergo careful ophthalmological assessment. Clinical evaluation alone may underestimate the injury, especially when swelling, pain, or superficial wounds obscure deeper structures. CT imaging should be considered the diagnostic standard in suspected facial fractures, orbital wall injuries, frontal sinus trauma, or possible intracranial extension. In selected cases, MRI may be useful for optic nerve, orbital apex, or soft tissue assessment, as illustrated by Al-Ghadeer et al. [20].

### 4.3. The Underestimated Risk of Unmounted Activities

One of the most important messages of this review is that unmounted equestrians and horse handlers represent a high-risk group for maxillofacial trauma. Public perception and preventive campaigns often focus on riding accidents and falls from horseback, but several included studies demonstrate that severe facial injuries frequently occur when the individual is on the ground.

The available evidence [4,5,13,15] consistently challenges the perception that ground-based interaction with horses is relatively safe. During grooming, feeding, shoeing, or routine handling, the individual may stand close to the hind limbs without using the protective equipment typically associated with riding. This combination of proximity, low helmet compliance, and limited facial protection creates a specific risk environment in which severe localized craniofacial injury may occur during otherwise ordinary activities.

This issue is also relevant in occupational settings. Farriers, hoof care practitioners, farm workers, and professional horse handlers may be repeatedly exposed to kicks and close-contact injuries. Fuhrer et al. [36] reported a substantial burden of head, face, and dental injuries among farriers and hoof care practitioners, while Cakabay et al. [34] identified horse kicks as a cause of farm-related maxillofacial trauma. Therefore, prevention should not be limited to recreational riders but should also involve occupational groups exposed to horses on a daily basis.

### 4.4. Clinical Implications for Emergency and Maxillofacial Management

The findings of this review support a mechanism-driven emergency and trauma approach to initial assessment, followed by targeted maxillofacial management. In patients presenting after a horse kick to the face, clinicians should assume a high-energy mechanism until proven otherwise. Because many patients initially present to emergency departments or trauma centers, the first priorities should be stabilization of life-threatening conditions, protection of the airway and cervical spine, assessment of neurological status, control of major hemorrhage, and identification of vision-threatening injuries before definitive fracture evaluation. The initial evaluation should not focus exclusively on visible lacerations or obvious fractures, but should systematically assess the orbit, globe, cranial vault, cervical spine, airway, occlusion, dentition, and soft tissues.

Sensory function in the infraorbital nerve distribution should be routinely documented in patients with orbital floor, zygomaticomaxillary complex, or midfacial fractures, as hypoesthesia may indicate involvement of the infraorbital canal or foramen. When a naso-orbito-ethmoid fracture is suspected, examination should also include medial canthal tendon stability, intercanthal distance, and the presence of traumatic telecanthus, because tendon disruption or detachment may substantially influence fracture classification and surgical reconstruction.

Imaging plays a central role. Multislice CT was emphasized by Kiuru et al. [26] for the acute assessment of serious horse-riding injuries, and CT remains essential for defining facial fracture patterns, orbital involvement, frontal sinus injury, skull base extension, and associated intracranial trauma. In horse-kick injuries involving the orbit or upper midface, the threshold for CT should be low, particularly when there is diplopia, visual impairment, periorbital hematoma, reduced ocular motility, infraorbital nerve symptoms, malocclusion, epistaxis, or neurological signs.

Airway management may be challenging in severe central facial injuries. Kannan et al. [17] described awake fiberoptic intubation guided by an intubating laryngeal mask in a patient with major midfacial disruption after horse kick. Although this is a single case report, it highlights a clinically important principle: bleeding, soft tissue distortion, disrupted nasal cavities, maxillary instability, and difficult positioning may make airway control complex. Early involvement of anesthesiology, maxillofacial surgery, and, when needed, otolaryngology or trauma surgery is advisable in severe midfacial trauma.

Multidisciplinary care is often required. Ocular injuries require ophthalmological assessment, frontal sinus and skull base injuries may require neurosurgical input, complex lacerations may require plastic surgical reconstruction, and pediatric mandibular fractures require consideration of growth and dentition. The cases described by Dunn et al. [18], Baugh et al. [32], Onișor-Gligor et al. [19], and Al-Ghadeer et al. [20] illustrate how horse-related craniofacial trauma can extend beyond routine fracture fixation.

Multidisciplinary management should be initiated early when the injury pattern extends beyond isolated facial fracture fixation. Maxillofacial surgeons are central to the assessment of facial skeletal stability, occlusion, dentition, and soft tissue injury, while ophthalmological involvement is essential in the presence of orbital trauma, visual impairment, globe injury, or suspected orbital compartment syndrome. Neurosurgical consultation may be required for skull base fractures, cerebrospinal fluid leakage, intracranial hemorrhage, frontal sinus posterior wall involvement, or penetrating craniofacial injury. Otolaryngology may contribute to airway management, severe epistaxis, sinonasal injury, and skull base assessment, whereas anesthesiology and trauma surgery are important for airway control, resuscitation, cervical spine protection, and associated multisystem injuries. Interventional radiology should also be considered in selected patients with persistent hemorrhage or suspected blunt cerebrovascular injury. Early communication among these specialties may facilitate prioritization of life-, vision-, and function-threatening conditions and improve the sequencing of definitive reconstruction.

**Figure 5:**
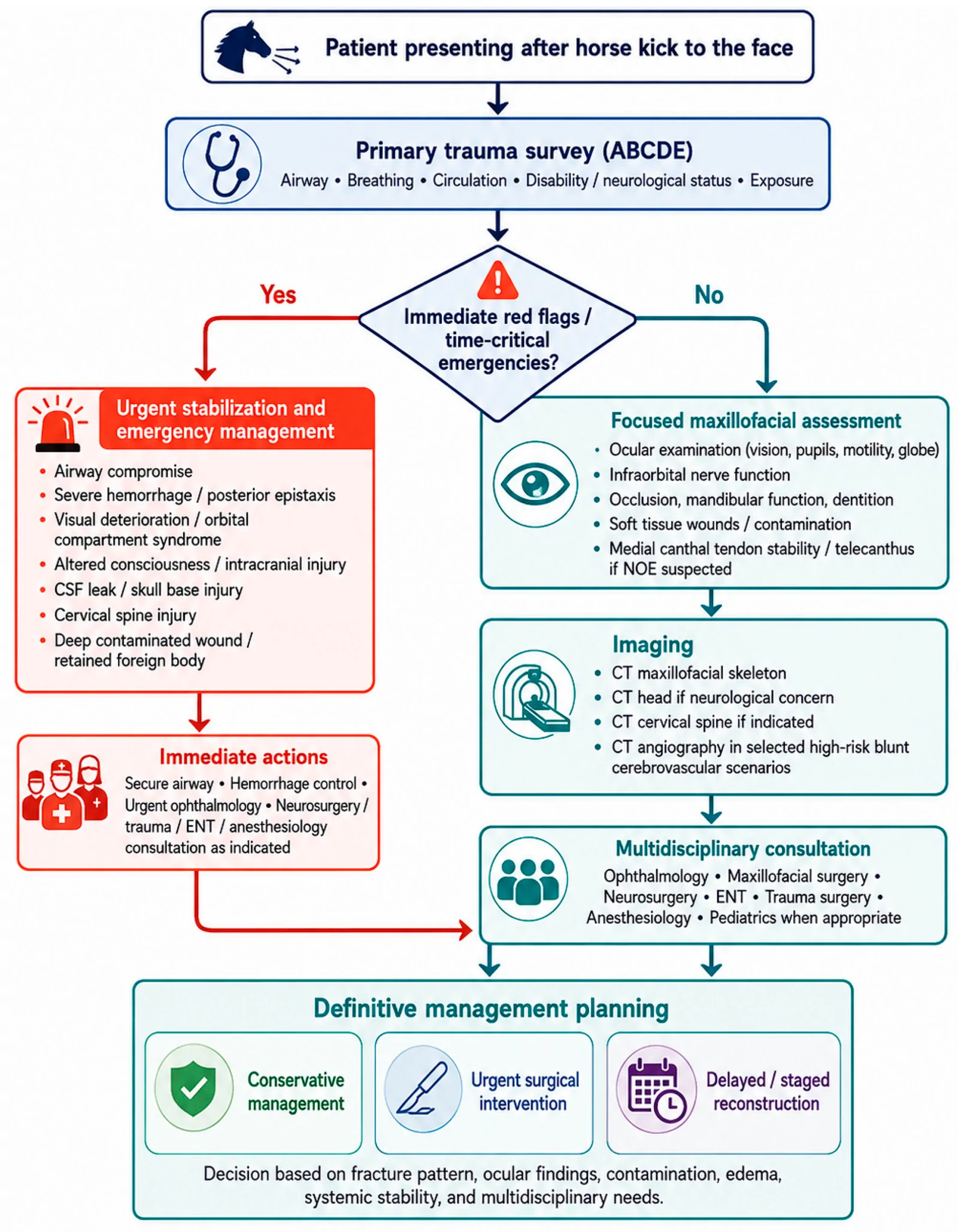
Proposed clinical management algorithm for patients presenting after horse-kick maxillofacial trauma.

To facilitate the practical application of these principles by emergency physicians, trauma teams, and maxillofacial surgeons, a proposed clinical management algorithm for the emergency assessment and early management of horse-kick maxillofacial trauma is summarized in Figure 5.

### 4.5. Time-Critical Maxillofacial Emergencies

From an emergency medicine perspective, the initial priority is not definitive fracture classification or fixation, but the rapid identification of life-, vision-, and neurological threats. Although most horse-related maxillofacial injuries are managed according to established trauma principles, direct hoof impact may produce time-critical conditions requiring recognition and treatment before definitive fracture reconstruction.

Airway compromise may occur in patients with severe mandibular or midfacial trauma. Bilateral mandibular body or parasymphyseal fractures, floor-of-mouth hematoma, posterior displacement of the tongue, active bleeding, soft tissue edema, and gross midfacial instability may progressively impair airway patency. Early assessment for voice changes, stridor, pooling of secretions, inability to tolerate the supine position, rapidly increasing swelling, and altered mental status is therefore essential. When airway difficulty is anticipated, early multidisciplinary planning involving anesthesiology, maxillofacial surgery, and, when appropriate, otolaryngology or trauma surgery is advisable. The severe midfacial injury described by Kannan et al. [17] illustrates the complexity of airway control in this setting.

Orbital compartment syndrome represents another vision-threatening emergency. Retrobulbar hemorrhage may cause rapidly increasing orbital pressure, proptosis, severe orbital pain, reduced visual acuity, a relative afferent pupillary defect, ophthalmoplegia, and a tense orbit. Because irreversible visual injury may develop rapidly, diagnosis is primarily clinical and should not be delayed by imaging when the findings are strongly suggestive. Immediate orbital decompression, generally through lateral canthotomy and cantholysis, should be considered while urgent ophthalmological support is obtained.

Major epistaxis may accompany complex midfacial, maxillary, or naso-orbito-ethmoid fractures. Initial management includes airway protection, hemodynamic assessment, direct pressure, topical vasoconstrictors, and appropriate nasal packing when not contraindicated. Persistent posterior bleeding may require endoscopic control, angiographic embolization, or operative management. Blind nasal instrumentation should be avoided when skull base injury is suspected.

Fractures involving the frontal sinus, central midface, or skull base should raise concern for intracranial extension and cerebrospinal fluid leakage. Clear rhinorrhea, otorrhea, pneumocephalus, neurological deterioration, or fractures involving the posterior frontal sinus wall or cribriform region warrant urgent neurosurgical assessment. Intracranial hemorrhage should also be excluded in patients with altered consciousness, focal neurological signs, severe headache, vomiting, or high-energy craniofacial injury.

Blunt cerebrovascular injury, including carotid or vertebral artery dissection, is uncommon but potentially catastrophic. It should be considered in patients with neurological deficits unexplained by initial brain imaging, cervical hematoma, severe cervical or skull base fractures, or high-energy facial trauma. In selected high-risk patients, computed tomography angiography may be indicated according to institutional trauma protocols.

Cervical spine injury must be considered during the initial assessment, particularly in patients with neurological symptoms, distracting facial injuries, altered consciousness, or evidence of associated multisystem trauma. Cervical spine precautions should be maintained until injury has been appropriately excluded.

Finally, contaminated open fractures and soft tissue wounds may require urgent exploration when retained foreign material, deep penetration, extensive devitalization, sinus communication, or intracranial extension is suspected. Early irrigation, conservative debridement, removal of foreign material, tetanus assessment, and appropriate antimicrobial management should precede definitive reconstruction when necessary. Small external wounds should not be assumed to represent limited injury, as illustrated by the orbitocranial retained foreign body reported by Dunn et al. [18].

### 4.6. Surgical and Conservative Treatment Considerations

The timing of definitive surgical treatment should be distinguished from the management of time-critical complications. Airway compromise, orbital compartment syndrome, uncontrolled hemorrhage, intracranial deterioration, and other immediately threatening conditions require urgent intervention and should not be delayed for definitive fracture planning. Once these emergencies have been addressed, the timing of reconstruction should be individualized according to neurological and systemic stability, fracture pattern, ocular findings, soft tissue condition, contamination, edema, and the need for multidisciplinary treatment. Early fixation may be appropriate when the patient is stable and the anatomy can be reliably restored, whereas delayed or staged reconstruction may be preferable in patients with severe swelling, extensive contamination, uncertain tissue viability, associated neurological injuries, or complex panfacial trauma. The available horse-specific literature does not provide sufficient comparative evidence to define precise timing thresholds for individual fracture patterns.

Treatment should be individualized according to fracture displacement, functional impairment, ocular involvement, dentition, age, contamination, and associated injuries. Conservative management remains appropriate in selected cases, particularly in minimally displaced fractures, pediatric mandibular trauma, and injuries where surgery would not improve the outcome. The case reported by Crean et al. [25], involving conservative management of bilateral mandibular fractures in a 2-year-old child, illustrates the importance of avoiding unnecessary surgical morbidity in early dentition when acceptable function and appearance can be achieved without fixation.

Operative treatment appears frequently necessary when horse-kick trauma produces displaced fractures, orbital volume changes, occlusal disturbance, mandibular instability, naso-orbito-ethmoid disruption, or complex soft tissue injury. The available evidence [5,13,14] does not support a single standardized operative strategy, because treatment is strongly influenced by anatomical pattern, contamination, associated injuries, patient age, and local expertise. The clinically relevant interpretation is therefore not the overall surgical rate, but the recurrent need for individualized reconstruction in injuries that combine skeletal disruption with ocular, neurological, airway, or soft tissue concerns.

Soft tissue management is another important point. Wounds caused by horse kicks may be contaminated by soil, hair, stable material, or foreign bodies. Dunn et al. [18] demonstrated that even a small periocular laceration may conceal a deep orbitocranial wooden foreign body. Therefore, wound exploration, irrigation, antibiotic consideration, tetanus status, and imaging should be carefully evaluated. In selected cases, retained foreign body, sinus involvement, or intracranial communication must be ruled out.

The timing of definitive soft tissue repair should be individualized according to the degree of contamination, tissue viability, crush injury, anatomical involvement, and soft tissue loss. Primary repair may be appropriate after meticulous irrigation, conservative debridement, removal of foreign material, and confirmation of adequate perfusion, particularly in facial wounds where early restoration of anatomical landmarks and functional structures is desirable. However, delayed closure or staged reconstruction should be considered when contamination is extensive, tissue viability remains uncertain, infection is already present, or repeated debridement is anticipated. In these cases, serial wound assessment may help define the extent of viable tissue before definitive reconstruction.

### 4.7. Outcomes, Morbidity, and Long-Term Sequelae

Although mortality appears uncommon in maxillofacial-specific series, the functional burden of horse-related facial trauma may be substantial [14,20,32,37]. Visual impairment, ocular motility disorders, malocclusion, sensory disturbance, neurological deficits, facial scarring, and the need for secondary procedures may persist beyond skeletal healing. The clinical significance of these injuries should therefore not be assessed solely according to fracture union, operative rates, or short-term survival, but also according to their potential long-term effects on vision, facial function, appearance, and quality of life.

The true magnitude of this morbidity is probably underestimated because follow-up duration, patient-reported outcomes, aesthetic results, sensory recovery, visual function, and quality of life were inconsistently documented across the included studies. Future research should therefore adopt standardized long-term outcome measures that capture functional, aesthetic, neurological, ophthalmological, and psychosocial consequences.

Mortality was rare and was mainly associated with severe head injury or multisystem trauma [1,11,14,26]. This reinforces the concept that maxillofacial injury after a horse kick may represent a marker of broader high-energy trauma and should prompt systematic assessment for intracranial, cervical, and systemic injuries.

### 4.8. Preventive Implications

The preventive message of this review is clear: current protective strategies are insufficient for the facial skeleton. Traditional helmets are essential for reducing cranial trauma, but they do not adequately protect the midface, orbit, mandible, or dentoalveolar structures. This limitation is particularly important in unmounted activities, where helmet use is often low and where the risk of facial impact from a kick may be high.

Several studies support this conclusion. Ueeck et al. [11] reported that most patients were not wearing helmets. Carmichael et al. [1] documented helmet use in only a small minority of admitted patients. Eckert et al. [16] reported that only one patient in the preliminary hoof-kick cohort was wearing a helmet. Maloney et al. [15] showed a striking difference in helmet compliance between mounted and unmounted patients, with much lower use during unmounted activities. Exadaktylos et al. [4] explicitly suggested that additional face shields may be protective.

Prevention should therefore move beyond the concept of “helmet use while riding” and include facial protection during high-risk ground activities. Faceguards, facial shields, protective eyewear, and possibly dental protection should be considered for selected groups, especially children, farriers, hoof care practitioners, stable workers, and individuals handling unfamiliar or poorly trained horses. Fuhrer et al. [36] specifically highlighted the occupational burden of head, face, and dental injuries among farriers and hoof care practitioners and emphasized the importance of eye protection and dental first aid.

Education is equally important. Many injuries occur during routine interactions with familiar horses, suggesting that risk may be underestimated during ordinary stable activities. Safety programs should emphasize safe positioning around horses, avoidance of standing directly behind the animal, recognition of behavioral warning signs, appropriate use of protective equipment, and supervision of children and inexperienced handlers. Riding schools, farms, stables, and professional equestrian environments should incorporate unmounted safety protocols, not only riding safety rules.

### 4.9. Future Research

Future studies should use standardized reporting frameworks for horse-related trauma. At minimum, authors should report whether the patient was mounted or unmounted, the exact mechanism of injury, the use of protective equipment, the anatomical distribution of fractures, ocular and neurological findings, treatment modality, complications, follow-up duration, and functional or aesthetic outcomes. Distinguishing between patient-level, fracture-level, and injury-level denominators is essential to improve comparability.

Prospective multicenter registries would be particularly valuable because individual centers usually collect small numbers of cases. Standardized data collection could allow more reliable estimates of the frequency of specific fracture patterns, surgical rates, ocular sequelae, and effectiveness of protective equipment. Research should also evaluate the biomechanical performance and user acceptability of facial protection systems, including faceguards and protective eyewear, especially during unmounted horse handling and farriery.

Finally, future studies should assess educational interventions aimed at reducing unmounted injuries. Because many severe facial injuries occur during routine handling rather than competitive riding, prevention programs should be designed for riders, children, stable workers, farriers, farmers, and recreational horse owners. The effectiveness of such interventions should be measured not only by reduction in injury incidence, but also by changes in protective equipment use and risk awareness.

### 4.10. Strengths and Limitations

This review has several strengths. It is, to our knowledge, one of the few systematic reviews specifically focused on horse-related maxillofacial trauma, with particular emphasis on horse-kick injuries. It integrates evidence from maxillofacial series, horse-kick-specific studies, ocular trauma studies, occupational cohorts, broader equestrian trauma registries, and severe case reports. This approach allowed identification of recurrent patterns across heterogeneous sources, particularly the association between horse kicks, unmounted activity, midface/orbital involvement, and need for preventive facial protection.

However, several limitations must be acknowledged. First, the available evidence is predominantly retrospective and observational, with relatively small sample sizes in maxillofacial-specific cohorts. Second, study populations were heterogeneous, including emergency department cases, trauma registry patients, operative-only cohorts, occupational groups, pediatric populations, and case reports. Third, denominators varied across studies: some reported data per patient, others per fracture, and others per injury subunit. This limited the feasibility of formal meta-analysis and required a primarily narrative synthesis. Moreover, because most of the available evidence consists of retrospective studies, small case series, and isolated case reports, the present review cannot establish definitive evidence-based recommendations regarding emergency assessment, surgical indications, treatment timing, or reconstructive strategy. The management considerations discussed in this review should therefore be interpreted as clinically oriented principles derived from the available literature and established trauma practice rather than as formally graded or validated guidelines.

Fourth, reporting of protective equipment, mounted versus unmounted status, follow-up duration, complications, and long-term outcomes was inconsistent. Fifth, broader equestrian trauma studies often grouped facial injuries with head, cervical spine, or ocular trauma, reducing their direct applicability to maxillofacial outcomes. Finally, case reports are subject to publication bias and tend to overrepresent rare or severe injuries. For this reason, they were used mainly to illustrate the clinical spectrum rather than to estimate frequency. Finally, case reports are subject to publication bias and tend to overrepresent rare or severe injuries. For this reason, they were used mainly to illustrate the clinical spectrum rather than to estimate frequency. Although the search combined four complementary bibliographic databases with manual reference-list screening, the absence of additional sources such as Embase and Google Scholar means that a small number of potentially relevant records may not have been identified.

## 5. Conclusions

Horse-related maxillofacial trauma is a clinically relevant injury pattern characterized by distinctive mechanisms and anatomical features. The principal finding of this review is that the horse kick should be regarded as a distinct localized high-energy blunt trauma mechanism rather than simply as one of several possible causes of equestrian injury. The concentrated transfer of kinetic energy from the hoof to a limited facial surface produces a recognizable injury pattern involving the midface, orbit, zygomaticomaxillary complex, mandible, and dentoalveolar region. Despite the localized nature of the impact, this mechanism may cause severe ocular, neurological, airway, vascular, and soft tissue complications and may also indicate associated intracranial or multisystem trauma.

Because of the localized high-energy nature of the mechanism, clinicians should maintain a high index of suspicion when assessing patients after horse-kick facial trauma, even when the visible external injury appears limited. Initial management should follow established emergency and trauma priorities, with stabilization of airway, breathing, circulation, neurological status, cervical spine, hemorrhage, and vision-threatening conditions before definitive maxillofacial reconstruction is planned. Early CT imaging, ophthalmological evaluation, airway planning, and multidisciplinary management should be considered according to injury severity and anatomical involvement. From a preventive perspective, conventional helmets are insufficient to protect the facial skeleton. Improved facial protection, targeted safety education, and specific prevention protocols for unmounted activities are needed to reduce the burden of horse-related maxillofacial injuries.

## Data Availability

No new original data were generated in this study. All data analyzed in this review were derived from previously published articles, which are cited in the reference list. Therefore, data sharing is not applicable to this article.

## Supplementary Materials

The following supporting information can be downloaded at https://www.mdpi.com/article/10.3390/jcm15166189/s1, Supplementary Document S1: Review protocol; Supplementary Document S2: PRISMA 2020 checklist; Table S1: Complete electronic search strategy; Table S2: Characteristics of the included studies.
