# Supplementary material for: The Impact of a Horse Kick: A Systematic Review of Horse-Related Maxillofacial Trauma
Source: J Clin Med. 2026 Aug 10;15(16):6189. doi: 10.3390/jcm15166189 (PMC13513070; doi:10.3390/jcm15166189)
Supplement: Supplementary file 1 [file jcm-15-06189-s001.zip › jcm-4483882-supplementary.pdf]

Table S1. Complete electronic search strategy

The Impact of a Horse Kick: A Systematic Review of Horse-Related Maxillofacial Trauma

Database / platform	Complete search string
**PubMed/MEDLINE**<br>National Library of Medicine	(<br>"Horses"[MeSH Terms] OR horse*[Title/Abstract] OR equine*[Title/Abstract]<br>OR equestrian*[Title/Abstract] OR "horse kick*"[Title/Abstract]<br>OR "hoof kick*"[Title/Abstract] OR "horse-related"[Title/Abstract]<br>OR "horse associated"[Title/Abstract] OR horseback[Title/Abstract]<br>)<br>AND<br>(<br>"Maxillofacial Injuries"[MeSH Terms] OR "Facial Injuries"[MeSH Terms]<br>OR "Orbital Fractures"[MeSH Terms] OR "Mandibular Fractures"[MeSH Terms]<br>OR "Tooth Injuries"[MeSH Terms] OR "maxillofacial injur*"[Title/Abstract]<br>OR "maxillofacial trauma*"[Title/Abstract] OR "craniofacial injur*"[Title/Abstract]<br>OR "craniofacial trauma*"[Title/Abstract] OR "facial injur*"[Title/Abstract]<br>OR "facial trauma*"[Title/Abstract] OR "facial fracture*"[Title/Abstract]<br>OR "maxillofacial fracture*"[Title/Abstract] OR "craniofacial fracture*"[Title/Abstract]<br>OR "mandibular fracture*"[Title/Abstract] OR "maxillary fracture*"[Title/Abstract]<br>OR "midface fracture*"[Title/Abstract] OR "zygomatic fracture*"[Title/Abstract]<br>OR "orbital fracture*"[Title/Abstract] OR "orbital injur*"[Title/Abstract]<br>OR "ocular injur*"[Title/Abstract] OR "eye injur*"[Title/Abstract]<br>OR "dentoalveolar trauma*"[Title/Abstract] OR "dental injur*"[Title/Abstract]<br>OR "tooth injur*"[Title/Abstract]<br>)
**Scopus**<br>Elsevier	TITLE-ABS-KEY(<br>horse* OR equine* OR equestrian* OR "horse kick*" OR "hoof kick*"<br>OR "horse-related" OR "horse associated" OR horseback<br>)<br>AND<br>TITLE-ABS-KEY(<br>"maxillofacial injur*" OR "maxillofacial trauma*" OR "craniofacial injur*"<br>OR "craniofacial trauma*" OR "facial injur*" OR "facial trauma*"<br>OR "facial fracture*" OR "maxillofacial fracture*" OR "craniofacial fracture*"<br>OR "mandibular fracture*" OR "maxillary fracture*" OR "midface fracture*"<br>OR "zygomatic fracture*" OR "orbital fracture*" OR "orbital injur*"<br>OR "ocular injur*" OR "eye injur*" OR "dentoalveolar trauma*"<br>OR "dental injur*" OR "tooth injur*"<br>)

Database / platform	Complete search string
**Web of Science Core Collection**<br>Clarivate	TS=(<br>horse* OR equine* OR equestrian* OR "horse kick*" OR "hoof kick*"<br>OR "horse-related" OR "horse associated" OR horseback<br>)<br>AND<br>TS=(<br>"maxillofacial injur*" OR "maxillofacial trauma*" OR "craniofacial injur*"<br>OR "craniofacial trauma*" OR "facial injur*" OR "facial trauma*"<br>OR "facial fracture*" OR "maxillofacial fracture*" OR "craniofacial fracture*"<br>OR "mandibular fracture*" OR "maxillary fracture*" OR "midface fracture*"<br>OR "zygomatic fracture*" OR "orbital fracture*" OR "orbital injur*"<br>OR "ocular injur*" OR "eye injur*" OR "dentoalveolar trauma*"<br>OR "dental injur*" OR "tooth injur*"<br>)
**Cochrane Library**<br>Search Manager	#1 MeSH descriptor: [Horses] explode all trees<br>#2 (horse* OR equine* OR equestrian* OR "horse kick*" OR "hoof kick*" OR "horse-related" OR "horse associated" OR horseback):ti,ab,kw<br>#3 #1 OR #2<br>#4 MeSH descriptor: [Maxillofacial Injuries] explode all trees<br>#5 MeSH descriptor: [Facial Injuries] explode all trees<br>#6 MeSH descriptor: [Orbital Fractures] explode all trees<br>#7 MeSH descriptor: [Mandibular Fractures] explode all trees<br>#8 MeSH descriptor: [Tooth Injuries] explode all trees<br>#9 ("maxillofacial injur*" OR "maxillofacial trauma*" OR "craniofacial injur*" OR "craniofacial trauma*" OR "facial injur*" OR "facial trauma*" OR "facial fracture*" OR "maxillofacial fracture*" OR "craniofacial fracture*" OR "mandibular fracture*" OR "maxillary fracture*" OR "midface fracture*" OR "zygomatic fracture*" OR "orbital fracture*" OR "orbital injur*" OR "ocular injur*" OR "eye injur*" OR "dentoalveolar trauma*" OR "dental injur*" OR "tooth injur*"):ti,ab,kw<br>#10 #4 OR #5 OR #6 OR #7 OR #8 OR #9<br>#11 #3 AND #10

Supplementary Table S2. Characteristics of the included studies

The Impact of a Horse Kick: A Systematic Review of Horse-Related Maxillofacial Trauma

Author, year	Country	Study design	Study period	Sample	Population	Main mechanism(s)	Main focus
**Kannan et al., 2000**	UK	Case report	N/A	1	Adult male with severe maxillofacial trauma	Horse kick	Airway management in severe facial injury
**Crean et al., 2000**	UK	Case report and literature review	N/A	1	2-year-old child	Horse kick	Pediatric mandibular fractures and conservative treatment
**Exadaktylos et al., 2002**	Switzerland	Retrospective ED database study	2000–2001	17	Unmounted equestrians with hoof-kick injuries	Horse kick	Hoof-kick injuries and prevention
**Kiuru et al., 2002**	Finland	Retrospective emergency radiology study	2000–2002	46	CT-assessed horse-riding trauma patients	Falls, kicks, crush injuries	Imaging findings in serious horse-riding accidents
**Ueeck et al., 2004**	USA	Retrospective Level I trauma center study	1998–2002	62	Patients with horse-related maxillofacial injuries	Falls, kicks, trampling	Patterns of horse-related maxillofacial trauma
**Yim et al., 2007**	Hong Kong	Retrospective trauma database study	2001–2005	36	Occupational/professional Jockey Club workers	Falls, kicks	Severe occupational horse-related trauma
**Fox et al., 2008**	USA	NEISS database analysis with illustrative cases	2005	1200	Emergency department equestrian injuries	Falls, stepped-on injuries, kicks, bites	Plastic surgery implications of equestrian injuries
**Cuenca et al., 2009**	USA	Retrospective pediatric study	11-year period	164	Pediatric equestrian trauma patients	Mainly falls/thrown; some kicked/trampled/trapped	Pediatric equestrian injuries
**Dunn et al., 2009**	USA	Case report	N/A	1	Adult equestrian	Horse kick	Orbitocranial penetrating trauma
**Antoun et al., 2011**	New Zealand	Retrospective maxillofacial fracture study	1996–2008	35	Unmounted equestrians with facial fractures	Kicks, head-butts, trampling	Maxillofacial fractures in unmounted equestrians
**Eckert et al., 2011**	Germany	Prospective multicenter study	2010	12*	Equestrians with horse-kick injuries	Horse kick	Prospective analysis of hoof-kick injuries
**Hasler et al., 2011**	Switzerland	Retrospective analysis and case-control survey	2000–2006; 2008	365; 61 cases/102 controls	Amateur equestrians	Falls, kicks, bites	Injury patterns and risk/protective factors
**Zhang et al., 2012**	China	Retrospective animal-related craniofacial injury study	1992–2008	149	Patients with animal-inflicted facial injuries	Bites, kicks, falls, trampling	Animal-related craniofacial trauma
**Baugh et al., 2013**	USA	Case report	N/A	1	Adult female ranch hand	Thrown from horse	Severe craniofacial polytrauma and epidural hematomas
**Carmichael et al., 2014**	USA	Retrospective Level I trauma registry study	2003–2007	284	Admitted horse-related trauma patients	Falls, kicks	Mounted versus unmounted injury patterns
**Islam et al., 2014**	Australia	Retrospective maxillofacial trauma study	5-year period	85	Patients with horse-related facial injuries	Falls, kicks, other mechanisms	Equine-associated maxillofacial injuries
**Srinivasan et al., 2014**	USA	Retrospective neurosurgical trauma series	2003–2011	80	Patients with neurological equestrian injuries	Kicked/stepped on, thrown/fell, horse fell on patient	Neurological injuries in equestrian sports

Author, year	Country	Study design	Study period	Sample	Population	Main mechanism(s)	Main focus
**Cakabay et al., 2016**	Turkey	Retrospective occupational/farm trauma study	2010–2012	146	Farmers with maxillofacial trauma	Cattle trauma, tools, tractor accidents, horse kicks	Farm-related maxillofacial trauma
**Weber et al., 2017**	International/Europe	Prospective trauma registry database study	1993–2012	679	Major equestrian trauma patients	Falls, kicks, crush injuries, carriage accidents	Mechanism-specific severe equestrian trauma
**Singleton et al., 2019**	Australia	Retrospective operative maxillofacial trauma series	2011–2016	28	Operatively managed equine-related maxillofacial trauma patients	Kicks, falls, head-butts	Operative management and fracture patterns
**Onişor-Gligor et al., 2019**	Romania	Case report and short literature review	N/A	1	Adult patient	Horse kick	NOE and frontal sinus fractures
**Al-Ghadeer et al., 2020**	Saudi Arabia	Case report	N/A	1	10-year-old boy	Horse kick	Traumatic optic nerve avulsion
**Diab and Moore, 2021**	Australia	Retrospective animal-related facial fracture study	2012–2017	50	Patients with animal-related facial fractures	Mainly horse-related kicking	Animal-related facial fractures
**Sritharan et al., 2022**	UK	Retrospective Level I trauma center study	2010–2020	51	Patients with horse-related maxillofacial injuries	Kicked/trodden, falls, head-butt, bite	Maxillofacial horse trauma
**Stier et al., 2022**	Germany	Retrospective maxillofacial fracture study	2000–2015	71	Patients with horse-related facial fractures	Mainly horse kicks	Fracture distribution, protective equipment, complications
**Fuhrer et al., 2023**	Switzerland	Nationwide questionnaire-based occupational survey	Not specified	278	Farriers and hoof care practitioners	Occupational head/face and dental injuries, mainly kicks	Occupational head, face, and dental injuries
**Maloney et al., 2025**	Ireland	Retrospective tertiary maxillofacial trauma review	2020–2025	73	Patients with equestrian-related facial fractures	Direct kicks, falls, other mechanisms	Equestrian-related maxillofacial fractures
**Moran et al., 2025**	USA	Retrospective ocular/adnexal trauma study	2015–2023	50	Patients with horse-related ocular/adnexal injuries	Kicks, falls, head-butt, buggy crash, trampling	Ocular and orbital trauma

Abbreviations: CT, computed tomography; ED, emergency department; N/A, not applicable; NEISS, National Electronic Injury Surveillance System; NOE, naso-orbito-ethmoid. *Preliminary cohort reported in the publication.

SUPPLEMENTARY DOCUMENT S1

Review Protocol

The Impact of a Horse Kick: A Systematic Review of Horse-Related Maxillofacial Trauma

Protocol status and provenance
This protocol was developed internally by the review team before completion of the review but was not prospectively registered in PROSPERO or another public registry. It is provided with the completed review to improve methodological transparency. Its publication as supplementary material should not be interpreted as prospective registration.

1. Administrative information

Item	Protocol information
**Review title**	The Impact of a Horse Kick: A Systematic Review of Horse-Related Maxillofacial Trauma
**Review type**	Systematic review with structured narrative synthesis
**Review framework**	PECO: population, exposure, comparator, and outcomes
**Registration**	Not registered
**Protocol document status**	Internal unregistered protocol reproduced as Supplementary Document S1
**Protocol coordination**	Luigi Angelo Vaira and Giacomo De Riu

Review team: Luigi Angelo Vaira; Hareem Qadeer; Sebastiano Stellino; Jerome R. Lechien; Antonino Maniaci; Fabio Maglitto; Giuseppe Consorti; Giulio Cirignaco; Łukasz Woźniak; Carlos Navarro-Cuéllar; Bożena Antonowicz; Jan Borys; Giovanni Salzano; Giacomo De Riu.

2. Background and rationale

Horse-related injuries occur during recreational, competitive, occupational, and routine ground-based activities. Falls from horseback are commonly emphasized in the general trauma literature, but direct horse kicks and hoof impacts can transmit a concentrated high-energy force to the face. The midface, orbit, zygomaticomaxillary complex, mandible, nasal region, and dentoalveolar structures may be affected, with associated ocular, neurological, airway, and multisystem injuries in severe cases.

The available evidence is dispersed across maxillofacial case series, trauma registries, equestrian injury cohorts, occupational studies, pediatric studies, ocular and neurological reports, and clinically important case reports. Differences in study populations, mechanisms, denominators, anatomical classifications, and outcome definitions limit direct comparison. A systematic review was therefore planned to identify recurring epidemiological and anatomical patterns, characterize management and outcomes, and identify opportunities for prevention, particularly during unmounted horse handling.

3. Objectives

The primary objective was to systematically identify and synthesize clinical evidence on horse-related maxillofacial trauma, with particular emphasis on horse-kick injuries.

Primary objectives: To describe the principal injury mechanisms; compare mounted and unmounted circumstances when reported; and characterize the anatomical distribution of facial, craniofacial, orbital, mandibular, maxillary, and dentoalveolar injuries.

Secondary objectives: To summarize associated injuries, imaging and airway considerations, operative and non-operative management, complications, functional or cosmetic outcomes, mortality, use of protective equipment, and preventive implications.

4. Review question and PECO framework

What mechanisms, injury patterns, management strategies, outcomes, and preventive factors are reported in patients sustaining horse-related maxillofacial trauma, particularly trauma caused by horse or hoof kicks?

PECO element	Prespecified definition
**Population**	Human patients of any age or sex with facial, craniofacial, orbital, ocular, dental, dentoalveolar, mandibular, maxillary, or maxillofacial trauma.
**Exposure**	Horse-associated trauma, including horse or hoof kicks, falls from horseback, trampling, crushing, head-butting, biting, stepping-on injuries, carriage-related events, and other direct horse-related mechanisms.
**Comparator**	When available: kick versus other mechanisms; mounted versus unmounted activity; protective equipment use versus non-use; operative versus non-operative management; and relevant demographic or occupational subgroups.
**Outcomes**	Mechanism, anatomical distribution, associated injuries, imaging, airway management, treatment, complications, hospital stay, functional and cosmetic outcomes, mortality, protective equipment, and prevention.

5. Eligibility criteria

Criterion	Prespecified eligibility rule
**Participants**	Human patients with extractable facial, craniofacial, orbital, ocular, dental, dentoalveolar, mandibular, maxillary, or maxillofacial injury data related to horses.
**Exposure and setting**	Mounted or unmounted horse-associated activities in recreational, competitive, occupational, agricultural, professional, or routine handling settings.
**Eligible designs**	Case reports, case series, retrospective or prospective observational studies, cohort studies, registry-based studies, trauma database analyses, and questionnaire-based studies containing original clinical data.
**Broader trauma cohorts**	Eligible only when relevant craniofacial, facial, orbital, ocular, dental, or maxillofacial data were separable or clinically informative. Studies without separable maxillofacial outcomes could be retained only for contextual discussion and not quantitative extraction.
**Exclusions**	Veterinary, cadaveric, experimental, biomechanical, or non-human studies; reviews, editorials, and letters without original clinical data; conference abstracts with insufficient extractable information; duplicate reports; and studies unrelated to facial or maxillofacial trauma.
**Participant restrictions**	No restriction by patient age or sex.
**Publication period**	No a priori publication-year restriction.

6. Information sources and search strategy

The planned electronic sources were PubMed/MEDLINE, Scopus, Web of Science Core Collection, and the Cochrane Library. Searches combined controlled vocabulary, where available, with free-text terms describing the equine exposure and the cranio-maxillofacial injury concept. Core exposure terms included horse, equine, equestrian, horse kick, hoof kick, horse-related, horse-associated, and horseback. Core outcome terms included facial trauma, maxillofacial trauma, craniofacial injury, facial fracture, mandibular fracture, orbital fracture, ocular injury, dental injury, and dentoalveolar trauma.

Search syntax was to be adapted to the field codes, controlled vocabulary, phrase searching, and truncation rules of each database. Full reproducible database-specific strings are reported separately in Supplementary Table S1. Reference lists of included articles and relevant reviews were to be screened manually to identify additional eligible reports.

7. Study records and selection process

Data management: Records retrieved from all databases were to be combined in a single review file and duplicates removed before screening. A standardized screening record was to document inclusion and exclusion decisions.

Title and abstract screening: Two reviewers were to screen titles and abstracts independently against the eligibility criteria. Records considered potentially eligible by either reviewer were to proceed to full-text assessment.

Full-text assessment: Two reviewers were to assess potentially eligible full texts independently. Reasons for exclusion were to be recorded at the full-text stage. Disagreements were to be resolved through discussion and consensus, with consultation of a third reviewer when necessary.

Study selection reporting: The final review was to report the numbers of records identified, deduplicated, screened, assessed in full text, excluded with reasons, and included in the qualitative synthesis using a PRISMA flow diagram.

8. Data extraction and data items

A standardized extraction form was planned and piloted on representative reports. Extraction was to be performed by members of the review team and checked for completeness and consistency. Ambiguities were to be resolved by consensus and, when necessary, by re-examination of the source article.

Study-level characteristics: First author, publication year, country, study design, study period, clinical setting, sample size, inclusion criteria, and principal focus.

Participant characteristics: Age, sex, occupational or recreational status, mounted or unmounted activity, and other relevant demographic information.

Exposure and mechanism: Horse kick or hoof impact, fall, trampling, crushing, stepping-on injury, head-butt, bite, horse falling on the patient, carriage event, or other reported horse-related mechanism.

Injury characteristics: Soft-tissue injury; fracture location and pattern; orbital, ocular, dental, dentoalveolar, mandibular, maxillary, midfacial, frontal sinus, or craniofacial involvement; and associated head, neurological, airway, or multisystem injuries.

Management and outcomes: Imaging, airway management, conservative treatment, operative fixation or reconstruction, multidisciplinary care, complications, revision procedures, follow-up, hospital stay, functional or cosmetic outcomes, visual outcomes, neurological sequelae, and mortality.

Prevention variables: Helmet use, facial protection, protective eyewear, activity at the time of injury, occupational context, supervision, and reported preventive recommendations.

9. Outcomes and prioritization

Primary outcomes: Distribution of injury mechanisms; mounted versus unmounted status; anatomical distribution of maxillofacial injuries; and proportion or description of patients requiring operative treatment when reported.

Secondary outcomes: Associated ocular, neurological, airway, and systemic injuries; complications; length of hospital stay; revision procedures; functional, visual, neurological, or cosmetic sequelae; mortality; protective equipment use; and preventive implications.

Unit of analysis: Patient-level data were preferred. When studies reported fractures or injury subunits rather than patients, the original denominator was to be retained and explicitly identified. Data with different denominators were not to be combined as though directly equivalent.

10. Methodological quality and risk of bias

Observational, retrospective cohort, and registry-based studies were to be assessed using the Newcastle–Ottawa Scale or an adapted version appropriate to the study design. Case reports and case series were to be assessed using the relevant Joanna Briggs Institute critical appraisal tools. Assessment domains included patient selection, clarity of exposure and mechanism reporting, outcome ascertainment, completeness of follow-up, comparability, denominator transparency, and overall reporting quality.

Risk-of-bias assessment was to be performed independently by two reviewers. Disagreements were to be resolved by consensus or, if needed, by a third reviewer. Operative-only cohorts and tertiary trauma-center series were to be interpreted with particular caution because of potential severity and referral bias.

11. Data synthesis

A structured narrative synthesis was planned as the primary analytical approach because substantial clinical and methodological heterogeneity was expected. Studies were to be organized by study type and clinical contribution, with maxillofacial-specific series and horse-kick-focused studies forming the core evidence base. Ocular, neurological, airway, pediatric, occupational, and broader equestrian trauma studies were to be used when they provided relevant extractable data or clinically important insights. Case reports were to be summarized separately as illustrations of rare or severe injury patterns rather than used to estimate frequency.

Planned synthesis domains: Mechanism of injury; mounted versus unmounted activity; anatomical injury pattern; associated trauma; management; complications and outcomes; protective equipment; and prevention.

Quantitative presentation: Study-level counts, proportions, means, medians, ranges, and other descriptive data were to be reported using the denominators supplied by each study. Particular care was planned where reports alternated between patient-level, fracture-level, and injury-subunit denominators.

Meta-analysis: Formal pooling was not planned as the primary approach because of anticipated heterogeneity in populations, settings, mechanisms, inclusion criteria, denominators, and outcome definitions. Exploratory pooling could be considered only if a sufficiently homogeneous group of studies and comparable outcome definitions were identified.

Subgroup comparisons: When data permitted, descriptive comparisons were planned for horse kicks versus other mechanisms, mounted versus unmounted injuries, adult versus pediatric populations, maxillofacial-specific versus broader trauma cohorts, and protective equipment use versus non-use.

12. Certainty of evidence and meta-biases

The certainty of evidence was to be assessed using the GRADE domains where applicable, including study limitations, inconsistency, indirectness, imprecision, and potential publication bias. Because the evidence base was expected to consist mainly of retrospective studies, case series, registry analyses, and case reports, the overall certainty was anticipated to be low to moderate.

Publication bias and selective reporting were to be considered qualitatively by examining the distribution of study designs, the prominence of severe or unusual cases, completeness of reported outcomes, and consistency between study methods and results. Funnel-plot assessment was not planned unless a sufficiently large and methodologically comparable set of studies became available.

13. Protocol amendments and deviations

Any substantive amendment to the eligibility criteria, outcomes, risk-of-bias approach, or synthesis plan was to be documented by the review team with its rationale. The decision to perform a structured narrative synthesis without formal meta-analysis was consistent with the prespecified approach for clinically and methodologically heterogeneous evidence. The inclusion of broader equestrian trauma studies for contextual discussion was permitted only when relevant maxillofacial information was clinically informative; studies without separable outcome data were not used for quantitative extraction.

14. Ethics, dissemination, funding, and conflicts of interest

Ethics: Ethics committee approval and individual informed consent were not required because the review used only data available in previously published reports and did not involve collection of new participant-level data.

Dissemination: The findings were planned for publication in a peer-reviewed journal, with the protocol, included-study characteristics, and complete electronic search strategy supplied as supplementary material.

Funding: No external funding was received for this review.

Conflicts of interest: The authors declared no conflicts of interest relevant to the review.

SUPPLEMENTARY DOCUMENT S2

PRISMA 2020 Checklist

The Impact of a Horse Kick: A Systematic Review of Horse-Related Maxillofacial Trauma

ì.

Section	Topic	Item #	Checklist item	Location where item is reported
TITLE	**Title**	1	Identify the report as a systematic review.	Title page.
ABSTRACT	**Abstract**	2	See the PRISMA 2020 for Abstracts checklist.	Abstract.
INTRODUCTION	**Rationale**	3	Describe the rationale for the review in the context of existing knowledge.	Section 1, paragraphs 1–5.
INTRODUCTION	**Objectives**	4	Provide an explicit statement of the objective(s) or question(s) the review addresses.	Section 1, final paragraph.
METHODS	**Eligibility criteria**	5	Specify the inclusion and exclusion criteria for the review and how studies were grouped for the syntheses.	Section 2, paragraphs 1, 3, 4, and 7; Supplementary Document S1, Sections 4–5.
METHODS	**Information sources**	6	Specify all databases, registers, websites, organisations, reference lists and other sources searched or consulted to identify studies. Specify the date when each source was last searched or consulted.	Section 2, paragraph 2; Supplementary Table S1.
METHODS	**Search strategy**	7	Present the full search strategies for all databases, registers and websites, including any filters and limits used.	Section 2, paragraph 2; Supplementary Table S1.
METHODS	**Selection process**	8	Specify the methods used to decide whether a study met the inclusion criteria of the review, including how many reviewers screened each record and each report retrieved, whether they worked independently, and if applicable, details of automation tools used in the process.	Section 2, paragraph 5; Supplementary Document S1, Section 7.
METHODS	**Data collection process**	9	Specify the methods used to collect data from reports, including how many reviewers collected data from each report, whether they worked independently, any processes for obtaining or confirming data from study investigators, and if applicable, details of automation tools used in the process.	Section 2, paragraph 6; Supplementary Document S1, Section 8.
METHODS	**Data items**	10a	List and define all outcomes for which data were sought. Specify whether all results that were compatible with each outcome domain in each study were sought and, if not, the methods used to decide which results to collect.	Section 2, paragraphs 1 and 6; Supplementary Document S1, Sections 8–9.
METHODS	**Data items**	10b	List and define all other variables for which data were sought. Describe any assumptions made about any missing or unclear information.	Section 2, paragraph 6; Supplementary Document S1, Section 8.
METHODS	**Study risk of bias assessment**	11	Specify the methods used to assess risk of bias in the included studies, including details of the tool(s) used, how many reviewers assessed each study and whether they worked independently, and if applicable, details of automation tools used in the process.	Section 2, paragraph 8; Supplementary Document S1, Section 10.
METHODS	**Effect measures**	12	Specify for each outcome the effect measure(s) used in the synthesis or presentation of results.	Section 2, paragraph 9; descriptive study-level counts and proportions were used. No pooled effect measure was calculated.
METHODS	**Synthesis methods**	13a	Describe the processes used to decide which studies were eligible for each synthesis.	Section 2, paragraphs 7 and 9; Supplementary Document S1, Section 11.
METHODS	**Synthesis methods**	13b	Describe any methods required to prepare the data for presentation or synthesis, such as handling of missing summary statistics or data conversions.	Section 2, paragraph 9; original study denominators were retained. No data conversion was performed.
METHODS	**Synthesis methods**	13c	Describe any methods used to tabulate or visually display results of individual studies and syntheses.	Section 2, paragraph 9; Figures 2–4; Supplementary Table S2.
METHODS	**Synthesis methods**	13d	Describe any methods used to synthesize results and provide a rationale for the choice(s). If meta-analysis was performed, describe the model(s), methods to identify statistical heterogeneity, and software used.	Section 2, paragraph 9; structured narrative synthesis was used because of substantial heterogeneity. No meta-analysis was performed.

Section	Topic	Item #	Checklist item	Location where item is reported
METHODS	Synthesis methods	13e	Describe any methods used to explore possible causes of heterogeneity among study results.	Section 2, paragraphs 7 and 9; descriptive comparisons by mechanism, mounted status, population, anatomical pattern, management, and outcome.
METHODS	Synthesis methods	13f	Describe any sensitivity analyses conducted to assess robustness of the synthesized results.	Not applicable; no meta-analysis or sensitivity analysis was conducted.
METHODS	Reporting bias assessment	14	Describe any methods used to assess risk of bias due to missing results in a synthesis arising from reporting biases.	Section 2, final paragraph; reporting bias and selective reporting were assessed qualitatively.
METHODS	Certainty assessment	15	Describe any methods used to assess certainty or confidence in the body of evidence for an outcome.	Section 2, final paragraph; GRADE domains were applied where appropriate.
RESULTS	Study selection	16a	Describe the results of the search and selection process, from the number of records identified to the number of studies included, ideally using a flow diagram.	Section 3, opening paragraph; Figure 2.
RESULTS	Study selection	16b	Cite studies that might appear to meet the inclusion criteria, but which were excluded, and explain why they were excluded.	No individually citable near-miss studies were identified; principal full-text exclusion reasons are reported in Section 3 and Figure 2.
RESULTS	Study characteristics	17	Cite each included study and present its characteristics.	Section 3.1; Supplementary Table S2; References 1, 2, 4–6, 11–20, and 25–37.
RESULTS	Risk of bias in studies	18	Present assessments of risk of bias for each included study.	Section 3.2; Figure 3.
RESULTS	Results of individual studies	19	For all outcomes, present for each study summary statistics for each group, where appropriate, and an effect estimate and its precision, ideally using structured tables or plots.	Sections 3.3–3.8 and Supplementary Table S2. Effect estimates with precision were not applicable to the heterogeneous narrative evidence base.
RESULTS	Results of syntheses	20a	For each synthesis, briefly summarise the characteristics and risk of bias among contributing studies.	Sections 3.1–3.8; Section 3.2 and Figure 3; Section 4.9.
RESULTS	Results of syntheses	20b	Present results of all statistical syntheses conducted. If meta-analysis was done, present the summary estimate, its precision, and measures of statistical heterogeneity.	Not applicable; no statistical synthesis or meta-analysis was performed.
RESULTS	Results of syntheses	20c	Present results of all investigations of possible causes of heterogeneity among study results.	Sections 3.3–3.8 and Sections 4.2–4.7; heterogeneity was explored descriptively.
RESULTS	Results of syntheses	20d	Present results of all sensitivity analyses conducted to assess the robustness of the synthesized results.	Not applicable; no sensitivity analysis was performed.
RESULTS	Reporting biases	21	Present assessments of risk of bias due to missing results arising from reporting biases for each synthesis assessed.	Section 3.2 and Section 4.9; reporting bias was considered qualitatively.
RESULTS	Certainty of evidence	22	Present assessments of certainty or confidence in the body of evidence for each outcome assessed.	Section 3.2 and Section 4.9; overall certainty was judged low to moderate.
DISCUSSION	Discussion	23a	Provide a general interpretation of the results in the context of other evidence.	Sections 4.1–4.7.
DISCUSSION	Discussion	23b	Discuss any limitations of the evidence included in the review.	Section 4.9.
DISCUSSION	Discussion	23c	Discuss any limitations of the review processes used.	Section 4.9.
DISCUSSION	Discussion	23d	Discuss implications of the results for practice, policy, and future research.	Sections 4.4–4.8 and Section 5.
OTHER INFORMATION	Registration and protocol	24a	Provide registration information for the review, including register name and registration number, or state that the review was not registered.	Supplementary Document S1, Protocol status and provenance and Section 1: review not registered.
OTHER INFORMATION	Registration and protocol	24b	Indicate where the review protocol can be accessed, or state that a protocol was not prepared.	Supplementary Document S1: Review Protocol.
OTHER INFORMATION	Registration and protocol	24c	Describe and explain any amendments to information provided at registration or in the protocol.	Supplementary Document S1, Section 13.

Section	Topic	Item #	Checklist item	Location where item is reported
**OTHER INFORMATION**	**Support**	**25**	Describe sources of financial or non-financial support for the review, and the role of the funders or sponsors in the review.	Funding statement: no external funding.
**OTHER INFORMATION**	**Competing interests**	**26**	Declare any competing interests of review authors.	Conflicts of Interest statement: no conflicts declared.
**OTHER INFORMATION**	**Availability of data, code and other materials**	**27**	Report which review materials are publicly available and where they can be found, including data collection forms, extracted data, analysis data, analytic code, and other materials.	Data Availability Statement; Supplementary Documents S1–S2 and Supplementary Tables S2–S3. No new original data or analytic code were generated.

Checklist source: Page MJ, McKenzie JE, Bossuyt PM, Boutron I, Hoffmann TC, Mulrow CD, et al. The PRISMA 2020 statement: an updated guideline for reporting systematic reviews. BMJ 2021;372:n71. The PRISMA 2020 checklist is licensed under CC BY 4.0.
